# Resonant breathing improves self-reported symptoms and wellbeing in people with Long COVID

**DOI:** 10.3389/fresc.2024.1411344

**Published:** 2024-07-12

**Authors:** Jessica Polizzi, Jenna Tosto-Mancuso, Laura Tabacof, Jamie Wood, David Putrino

**Affiliations:** Cohen Center for Recovery from Complex Chronic Illnesses, Icahn School of Medicine at Mount Sinai, New York, NY, United States

**Keywords:** Long COVID, resonant breathing, autonomic nervous system, autonomic dysfunction, rehabilitation

## Abstract

**Introduction:**

Long COVID involves debilitating symptoms, many of which mirror those observed with dysautonomia, and care must be taken with rehabilitation for autonomic dysfunction to avoid post-exertional malaise/post-exertional symptom exacerbation. Resonant breathing (breathing slowly at a defined rate of breaths per minute) requires less exertion and can potentially improve autonomic function. The objective of this work was to report on the impact of a resonant breathing program on self-reported symptoms and wellbeing in people with Long COVID.

**Methods:**

A retrospective analysis of de-identified data was completed in a convenience sample of people with Long COVID, who participated in the Meo Health (formerly known as Stasis HP) resonant breathing program. Participants completed baseline and follow up surveys.

**Results:**

Data were available for 99 participants. Most measures of symptoms and wellbeing improved at follow up, with the largest differences per participant seen in sense of wellness (47.3%, *p* < 0.0001), ability to focus (57.5%, *p* < 0.0001), ability to breathe (47.5%, *p* < 0.0001), ability to control stress (61.8%, *p* < 0.0001) and sleep quality (34.9%, *p* = 0.0002). Most (92%) participants reported improvement at follow up on the Patient Global Impression of Change Scale.

**Conclusion:**

Self-reported symptoms and wellbeing improved in people with Long COVID completing resonant breathing. Resonant breathing can be considered as an option within the broader treatment plan of people with Long COVID.

## Introduction

Post-acute sequelae of SARS-CoV-2 infection (PASC), also known as Long COVID, is a post-acute infection syndrome that involves debilitating symptoms and affects at least 6% (almost 20 million) of the adult US population (1). These persistent symptoms include post-exertional malaise, fatigue, weakness, pain, shortness of breath, cognitive dysfunction, sleep disturbances, fevers, and gastrointestinal issues, and affect every organ system (2–4). Further, many symptoms of Long COVID mirror those observed with dysautonomia, including orthostatic intolerance, palpitations, tachycardia, syncope, labile blood pressure, dizziness, and exercise intolerance (5).

Orthostatic intolerance and postural orthostatic tachycardia syndrome (POTS) can commonly follow acute viral infections, and are similarly observed in up to 80% of people with Long COVID (5–8). Prior to the COVID-19 pandemic, approximately 50% of individuals with POTS reported a history of viral infection prior to symptom onset, suggesting this as a potential trigger (9). The presence of autonomic dysfunction in Long COVID is possibly due to mechanisms such as viral- or immune-mediated disruption of the autonomic nervous system (10), brainstem signaling abnormalities (11), and/or small fiber neuropathy (12). In addition, sympathetic mediated hypocapnia has been reported in people with autonomic dysfunction (13), and in Long COVID in the absence of hyperventilation (14), with an overlap observed in the symptoms of hypocapnia and Long COVID.

While autonomic rehabilitation can be of benefit to some people with Long COVID, care must be taken to avoid post-exertional malaise/post-exertional symptom exacerbation (PEM/PESE) (6). Encouraging people with Long COVID to engage in physical activity that exceeds their exertional tolerance has been a commonly identified cause of worsening symptoms in self-report surveys (2, 3). Furthermore, there is evidence to suggest that overexertion of people with Long COVID who have PEM/PESE can lead to local and global physiological damage (15). As such, strategies that may regulate autonomic nervous system activity in Long COVID with minimal exertion should be investigated for feasibility and tolerability. Resonant breathing (breathing slowly at a defined rate of breaths per minute) requires less exertion than current approaches for autonomic rehabilitation (most commonly graded exercise therapy (16), and have previously been demonstrated to alleviate hypocapnia and balance sympathetic and parasympathetic activation (17). The physiological benefit of resonant breathing is underpinned by increased responsiveness of the baroreflex (18, 19), leading to improved homeostatic control of blood pressure and heart rate, alongside increased parasympathetic activation (17). Heart rate variability (HRV), an indirect measure of autonomic regulation, is decreased (i.e., worse) in people with Long COVID and is associated with increased self-reported fatigue (20). Resonant breathing could theoretically serve to counterbalance this abnormally increased sympathetic nervous system activity and autonomic dysfunction occurring in Long COVID (21).

Consensus guidelines for the treatment of autonomic dysfunction in Long COVID encourage clinicians to identify and address the most disabling symptoms (6, 22). Given the potential ability of resonant breathing exercises to improve the function of the autonomic nervous system, including this as part of the broader approach to autonomic rehabilitation has merit. The primary objective of this work was to report on the impact of an online resonant breathing program on self-reported symptoms and wellbeing in people with Long COVID.

## Methods

### Study design

This was a retrospective analysis of de-identified data from a convenience sample of people with Long COVID, who participated in the Meo Health (formerly known as Stasis HP) resonant breathing program between September 2020 and November 2021. This study was determined to be exempt from the need for ethics review by the Mount Sinai Program for the Protection of Human Subjects due to the de-identified nature of the data (STUDY-24-00246).

### Participants

People aged 18 years or older with Long COVID were either referred to the Meo Health program via their care provider, or self-referred to the program. Participants were included in the analysis if they completed at least 4 weeks of the Meo Health program and completed both baseline and follow up surveys. All participants had confirmed (by polymerase chain reaction (PCR) and/or antibody test) or probable (diagnosed by a medical doctor in accordance with World Health Organization recommendations (23) previous COVID-19 infection and diagnosis of Long COVID (defined as experiencing symptoms >12 weeks since initial symptom onset). There were no specific exclusion criteria.

### Resonant breathing protocol

Participants underwent an initial 4-week progressive resonant breathing program using a 4:6 (inspiratory:expiratory) second cadence. Participants were instructed via recorded video content, and advised to complete the resonant breathing exercises twice per day for five days per week (upon waking and in the evening prior to sleep). Instructions for nasal unblocking were also provided. The time of the sessions were initially set at 10 min in length, increasing up to 30 min by the end of week four as tolerated. Participants were advised to avoid caffeine or other stimulants prior to the sessions. All participants had the option to request an online supervised session to check technique, but this was not mandatory. At onboarding, participants were encouraged to continue the program beyond the initial 4 weeks (for 12 weeks and beyond), as a mainstay part of their broader Long COVID treatment plan. No other treatment was provided as part of the Meo Health program.

### Outcome survey

A survey designed for the program by Meo Health was administered prior to commencement and at a follow up date according to the length of time the participant used the program. The survey collected basic demographic data (gender and age), and self-reported symptoms and wellbeing on a Likert scale (1–5, 1 = Very low, 5 = Very high), including stress, ability to control stress, anxiety, sleep quality, breathlessness, ability to breathe, fatigue, ability to focus, and sense of wellness. The first survey also included self-reported results from the max exhale test (MET) (amount of time it takes the participant to exhale as slowly as possible after full nasal inspiration) and breath hold test (BHT) (time able to hold breath after normal inhalation), while the follow up survey included the Patient Global Impression of Change (PGIC), and how many times per week and minutes per session the resonant breathing was performed.

### Statistical analysis

Statistical analyses were undertaken with Stata (StataCorp, Stata Statistical Software Release: V.14). Data were analyzed using descriptive statistics, *t*-tests pairwise correlation and linear regression. Categorical data are presented as frequencies and proportions. Continuous data are presented as mean and standard deviation (SD), median and interquartile range (IQR) or median and range. Bonferroni's adjustment was applied post *t*-tests (*n* = 9) to provide a corrected alpha of 0.0056. Level of significance was set to 0.05 for all other tests.

## Results

Data were available for 99 participants [78 (78%) female, aged median (IQR) 49 (41–57) years] who were enrolled in the program for at least 4 weeks and completed both baseline and follow up surveys. The median time at follow up was 95 (29–378) days. Baseline MET was 28 (16–47) s, and BHT was 41 (28–50) s. Participants reported completing the resonant breathing program 7 (5–8) times per week, for 8 (8–13) min per session.

**Figure 1:**
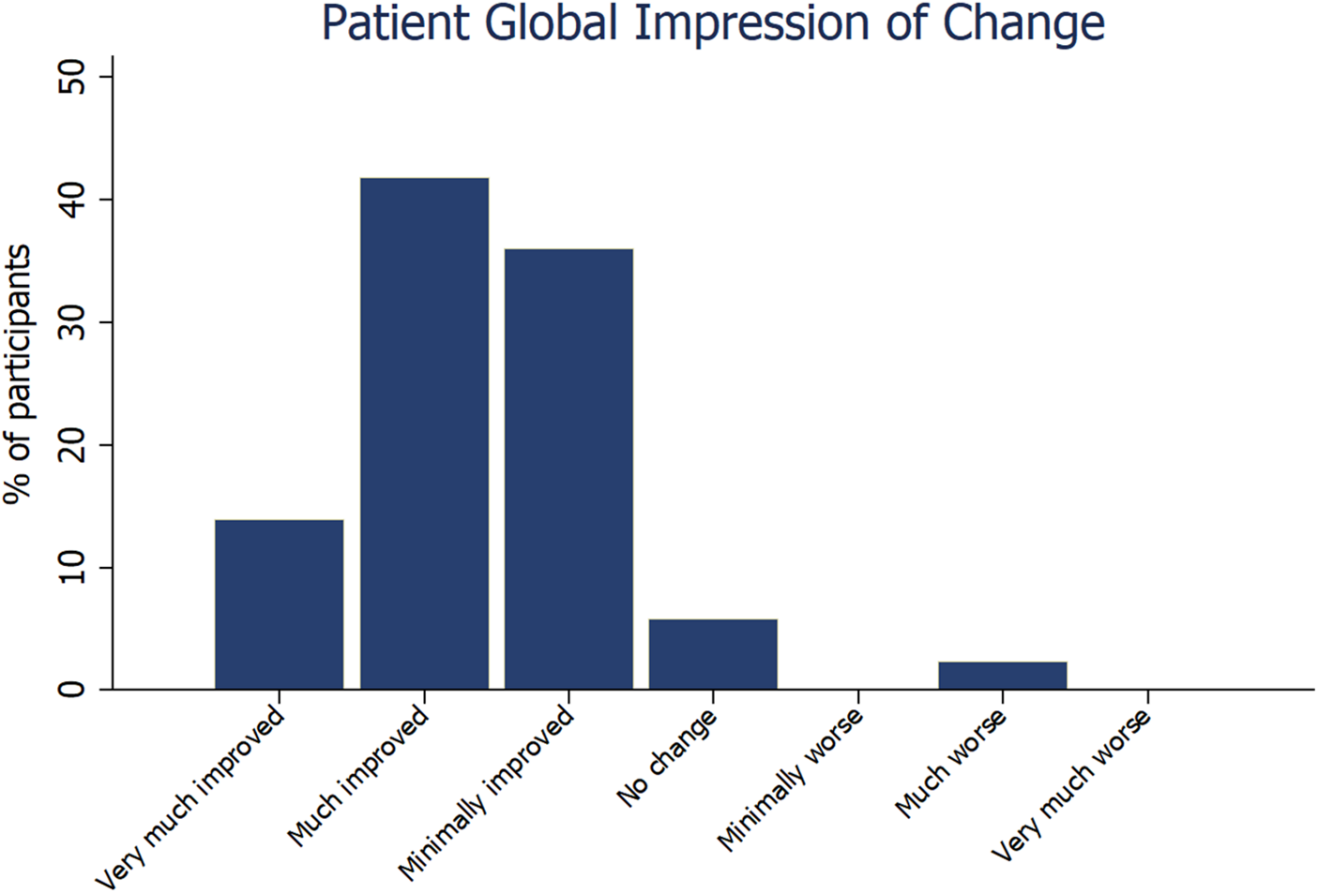
Patient global impression of change scores for all participants (*n* = 99).

Nearly all (89%) measures of symptoms and wellbeing improved at follow up, with the largest mean (SD) percentage improvement per participant observed in ability to control stress [61.8 (96.0)%, *p* < 0.0001], ability to focus [57.5 (76.3)%, *p* < 0.0001], ability to breathe [47.5 (82.3)%, *p* < 0.0001], sense of wellness [47.3 (67.5)%, *p* < 0.0001], and sleep quality [34.9 (72.5)%, *p* = 0.0002] (Table 1). Most (92%) participants reported some level of improvement at follow up on the Patient Global Impression of Change Scale (Figure 1). The median time at follow up was not a predictor of changes in any outcomes. Improvement in ability to control stress was positively correlated with the number of times the Meo Health program was used each week (*r* = 0.38; *p* < 0.001). Improvement in ability to control stress was also associated with reduced self-reported anxiety (*r* = −0.29; *p* = 0.003) and improved sleep (*r* = 0.30; *p* = 0.003).

**Table 1:** Self-reported symptoms and wellbeing for all participants (*n* = 99).

	Baseline	Follow up	Mean diff. (95% CI)	Change per participant (%)	*P*-value*
Stress	3.25 (1.01)	2.95 (0.97)	−0.29 (−0.57 to −0.02)	−2.7 (37.9)	0.0389
Ability to control stress	2.39 (1.09)	3.16 (0.97)	0.77 (0.50–1.06)	61.8 (96.0)	<0.0001
Anxiety	3.62 (1.11)	2.66 (0.98)	−0.61 (−0.90 to −0.32)	−9.5 (48.0)	0.0001
Sleep quality	2.81 (1.02)	3.35 (0.97)	0.55 (0.27–0.82)	34.9 (72.5)	0.0002
Breathlessness	2.67 (1.03)	2.09 (1.05)	−0.57 (−0.87 to −0.28)	−14.1 (45.8)	0.0001
Ability to breathe	2.81 (0.95)	3.61 (0.90)	0.80 (0.54–1.06)	47.5 (82.3)	<0.0001
Fatigue	3.85 (1.12)	3.28 (1.04)	−0.57 (−0.87–0.26)	−3.8 (62.8)	0.0003
Ability to focus	2.53 (0.94)	3.45 (0.82)	0.93 (0.68–1.18)	57.5 (76.3)	<0.0001
Sense of wellness	2.14 (0.86)	2.79 (0.80)	0.65 (0.41–0.88)	47.3 (67.5)	<0.0001

Data are presented as mean (SD) unless otherwise stated.

* Bonferroni's adjustment was applied post *t*-tests (*n* = 9) to provide a corrected alpha of 0.0056.

## Discussion

The emergence of Long COVID has presented significant challenges to healthcare and the scientific community, with no approved treatments currently available (pharmacological or non-pharmacological). These data provide insight into the potential role of resonant breathing in the alleviation of some of the symptoms of Long COVID, alongside an ability to improve self-reported measures of wellbeing in a small cohort.

The positive outcomes observed in people with Long COVID performing at least 4 weeks of resonant breathing support the hypothesis of improved autonomic function as the possible mechanism, aligning with previous findings highlighting the potential role of breathwork as a therapy to address autonomic dysfunction in Long COVID (24) and other conditions (25–27). Controlled breathing may improve autonomic function via improving the baroreflex (18, 19), enhancing parasympathetic activity and mitigating stress responses (28). Symptoms of autonomic dysfunction feature heavily in many presentations of Long COVID (2, 3, 10), therefore the reported data are encouraging. Resonant breathing is a relatively simple technique that can be easily integrated into the treatment programs of people with Long COVID.

In addition, using HRV biofeedback may further optimize the benefits of resonant breathing (29), warranting further investigation. HRV biofeedback can be integrated into smartphone applications and is therefore accessible for use by people in the home setting. The feasibility of using HRV biofeedback has been demonstrated in a small number (13) of people with Long COVID, with observed improvements in measures of symptoms and wellbeing (21). Home-based rehabilitation programs are feasible in people with Long COVID (30, 31), and resonant breathing could be implemented remotely to reduce the burden on the patient. The broader rehabilitation program for people with Long COVID should address all potential sources of symptomatology, including autonomic (6), cardiovascular (32), pulmonary (33), neurological (34), cognitive (35) and musculoskeletal (36). As resonant breathing may be useful to assist with symptom stabilization, potential synergistic effects with other treatment approaches should be investigated.

The improvements in outcomes including stress, focus, breathing, wellness and sleep mirror what has been reported in other groups performing resonant breathing, with evidence suggesting that this technique has a range of positive effects on stress reduction, mood regulation, awareness, and interoceptive sensitivity across various populations (37–40). In addition to benefits in autonomic function, resonant breathing may help attenuate some of the feelings of anxiety associated with living with a severe complex chronic illness of proven immunological origin (4). The association between frequency of weekly use of the Meo Health program and improvements in ability to control stress, with a subsequent association with improvements in self-reported anxiety, supports the need for consistency in performing a resonant breathing program to achieve the desired results within this domain.

The majority of participants in this cohort were female (78%) which aligns with other studies reporting a bias towards Long COVID affecting women disproportionately (1–3). The age of participants in the present study was also similar to these cohorts. Long COVID affects more females possibly due to differences in hormones (41) as well as the ability of women to mount a faster and larger immune response (42), which may result in the longer-term symptoms experienced.

There are several limitations to be considered with this report. The data were analyzed retrospectively from a small cohort of people with Long COVID, with no prospective study design or control group. Information regarding how long each participant had been experiencing symptoms of Long COVID was not obtained, making it difficult to determine any level of benefit relating to how soon resonant breathing was commenced after initial infection. Further, while it is presumed that the participants were completing the program consistently up until the time at follow up, the data are self-reported and there may be discrepancies between responses and actual rates of adherence to the program. Other therapies being used were not considered and therefore could not be controlled for in the analysis, and there is a possibility that some participants experienced improvements in their outcomes spontaneously over time; however, recovery from Long COVID can be relatively uncommon during this timeframe, with 85% still reporting symptoms at one year (43). Obtaining objective measures of autonomic function was also beyond the scope of this study, and therefore any suspected improvements could not be quantified and reported.

## Conclusion

This study provides some insight into the potential role and benefit of resonant breathing, and its feasibility as a simple and easy to use non-pharmacological treatment option for Long COVID. Long COVID impacts multiple body systems and requires a multi-targeted treatment approach (44), and a low-exertion therapy for improving self-reported symptoms and wellbeing has the potential to be of benefit for many people with Long COVID. These results now need to be reproduced in larger prospective studies with adequate controls, appropriate testing of autonomic function, and with sufficient power to explore the relationships between age, sex and other variables on outcomes.

## Data Availability

The datasets presented in this article are not readily available because data will only be made available via reasonable request to the corresponding author. Requests to access the datasets should be directed to David Putrino, david.putrino@mountsinai.org.
